# Gentiolactone, a Secoiridoid Dilactone from *Gentiana triflora*, Inhibits TNF-α, iNOS and Cox-2 mRNA Expression and Blocks NF-κB Promoter Activity in Murine Macrophages

**DOI:** 10.1371/journal.pone.0113834

**Published:** 2014-11-25

**Authors:** Hidetoshi Yamada, Sayaka Kikuchi, Tomoki Inui, Hideyuki Takahashi, Ken-ichi Kimura

**Affiliations:** 1 Department of Bioresource Sciences, Iwate Biotechnology Research Center, Kitakami, Iwate, Japan; 2 Graduate School of Agriculture, Iwate University, Morioka, Iwate, Japan; Universidade de São Paulo, Brazil

## Abstract

**Background:**

Gentian roots have been used as a herbal medicine because of their anti-inflammatory activities. However, the molecular mechanisms of these anti-inflammatory effects remain to be completely explained.

**Methods and Findings:**

Here, we investigated anti-inflammatory effects of gentian roots and showed that root extracts from *Gentiana triflora* inhibited lipopolysaccharide (LPS)-induced expression of TNF-α in RAW264.7 cells. The extracts also contained swertiamarin and gentiopicroside, which are the major active compounds of gentian roots; however, neither compound had any effect on LPS-induced TNF-α production in our test system. We isolated gentiolactone as an inhibitor of TNF-α production from the extracts. Gentiolactone also inhibited LPS-induced inducible nitric oxide synthase (iNOS) and cyclooxygenase-2 (Cox-2) expression at the mRNA level. Moreover, gentiolactone suppressed NF-κB transcriptional activity without inhibition of IκB degradation or NF-κB nuclear transport.

**Conclusions:**

Our results indicate that inhibition of TNF-α, iNOS and Cox-2 expression by gentiolactone is one of the mechanisms of the anti-inflammatory properties of gentian roots.

## Introduction

Gentians (*Gentiana* spp.) are alpine plants whose roots and rhizomes have been used as herbal medicines to treat gastrointestinal disorders and inflammatory diseases. In *Gentiana lutea*, gentiopicroside is the most abundant active compound followed by loganic acid, swertiamarin and the xanthone glycosides [1]. Gentiopicroside and swertiamarin are secoiridoid glycosides, which are reported to have antimicrobial [2], antifungal [3], hepatoprotective [4], and antilipidaemic activities [5],[6]. Although numerous studies have been performed on the anti-inflammatory effects of gentian root extracts [7],[8], most investigations did not provide any data on the underlying molecular mechanisms.

Oxidative stress and inflammation are crucial for defense against infections, but they can initiate a number of deleterious effects, such as impairment of intracellular calcium transport and production of cytokines. In chronic inflammatory diseases, such as rheumatoid arthritis, overexpression of the pro-inflammatory cytokine tumor necrosis factor α (TNF-α) is thought to play an important role in disease progression [9],[10]. Inducible nitric oxide synthase (iNOS) is a pro-inflammatory enzyme that is involved in the production of nitric oxide (NO). In chronic kidney disease, oxidative stress has been found to increase in parallel with diseases progression [11]. The pro-inflammatory enzyme cyclooxygenase-2 (Cox-2) is induced mainly at sites of inflammation in response to inflammatory stimuli including TNF-α. Elevated Cox-2 expression has been found in approximately 50% of adenomas and 85% of adenocarcinomas [12].

Members of the Rel/NF-κB family of transcription factors are key regulators of innate and adaptive immune responses and several cellular processes, including survival, growth, and proliferation [13],[14]. In mammals, the Rel family consists of five members, c-Rel, p65 (RelA), RelB, NF-κB1 (p50/p105), and NF-κB2 (p52/p100), that bind DNA as homodimers and heterodimers through a conserved N-terminal Rel homology region (RHR) [15]. Rel dimers are often maintained in an inactive state by cytoplasmic association with IκB proteins [16]. Cell activation leads to signal transduction cascades that result in the phosphorylation and degradation of the IκBs, allowing rapid nuclear translocation and DNA binding of the Rel dimers.

The present study was aimed at elucidating the molecular basis of the anti-inflammatory effect of roots of the Japanese gentian, *G. triflora*. We found that a methanol extract of *G. triflora* roots inhibited LPS-induced expression of TNF-α, iNOS, and Cox-2 in RAW264.7 cells. Furthermore, activity-guided fractionation of root extracts identified gentiolactone as the active compound: TNF-α expression was inhibited by gentiolactone but not by gentiopicroside or swertiamarin. These results indicate that inhibition of TNF-α, iNOS, and Cox-2 expression by gentiolactone is one of the mechanisms involved in the anti-inflammatory properties of *G. triflora* roots.

## Results

**Figure 1:**
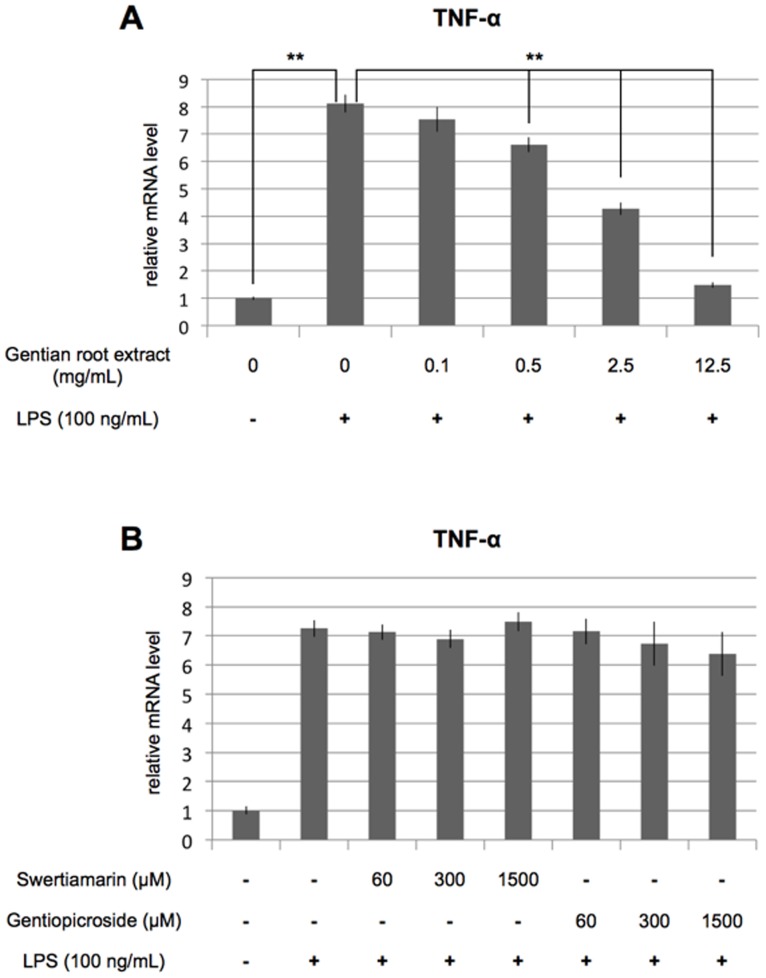
MeOH extract from gentian roots inhibits LPS-induced TNF-α production. RAW264.7 cells were cultured for 4 hours with LPS (100 ng/mL) and 0.1, 0.5, 2.5 or 12.5 mg/mL MeOH extract (A), or 60, 300 or 1500 µM swertiamarin or gentiopicroside (B). TNF-α mRNA levels were measured by real-time PCR. Plotted values represent the mean value ± SD from four independent cultures. *p<0.05, **p<0.01.

To examine the anti-inflammatory activity of gentian root extracts, we investigated their ability to inhibit LPS-induced expression of TNF-α in RAW264.7 cells. TNF-α mRNA expression was significantly up-regulated at 4 hours after treating the cells with LPS. This upregulation was inhibited in a concentration-dependent manner by the root extracts (Fig. 1A).

Gentiopicroside and swertiamarin have been reported to be the major active compounds of gentian roots. We measured the concentrations of gentiopicroside and swertiamarin in 12.5 mg/mL MeOH extracts using LC/TOFMS and found 1.41 mM and 0.16 mM, respectively. On the basis of these concentrations, we set the highest concentration of gentiopicroside and swertiamarin to be tested at 1.5 mM and examined the inhibitory effects on LPS-induced TNF-α expression. Neither compound showed any inhibitory effect (Fig. 1B).

**Figure 2:**
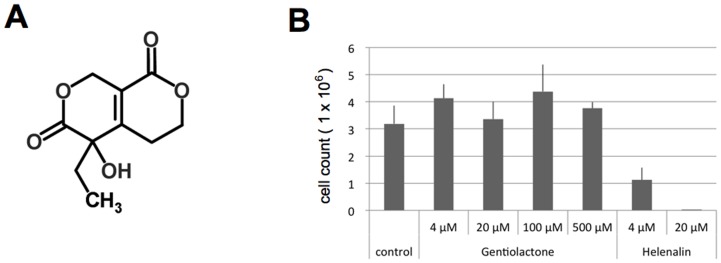
Gentiolactone and cytotoxicity of gentiolactone. A, Structure of gentiolactone. The molecular formula of gentiolactone is C_10_H_12_O_5_ and its monoisotopic mass is 212.068. B, RAW264.7 cells were seeded in 6-well culture plates (1×10^6^ cells/well) and cultured with gentiolactone or helenalin for 24 hours. At the end of this period, the number of living cells in each well was counted.

**Figure 3:**
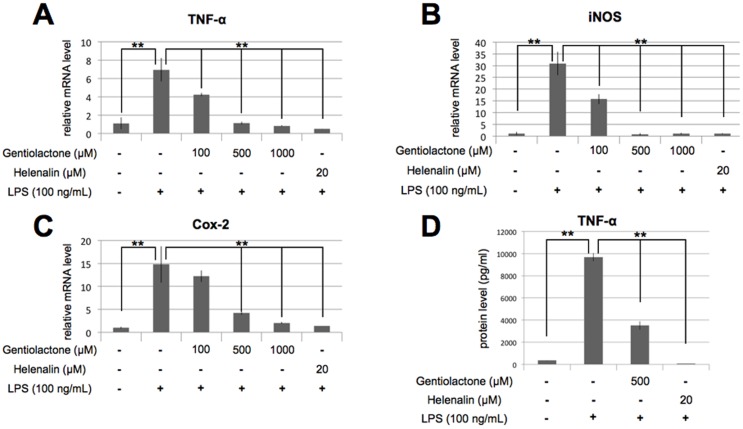
Gentiolactone inhibits LPS-induced TNF-α mRNA expression. RAW264.7 cells were cultured for 4 hours with LPS (100 ng/mL) and 100, 500 or 1000 µM gentiolactone. The mRNA levels of TNF-α (A), iNOS (B) and Cox-2 (C) were measured by real-time PCR. (D) TNF-α protein levels were measured with an ELISA assay. Plotted values represent the mean value ± SD from four independent cultures. *p<0.05, **p<0.01. Helenalin was used as a positive control.

We purified the active compound that inhibited LPS-induced TNF-α mRNA expression from gentian root extracts using the inhibitory activity-guided fractionation method (Fig. S1). The major compound in the most active fraction was identified by LC-TOFMS and NMR as gentiolactone (Fig. 2A). To examine its cytotoxicity, we cultured RAW264.7 cells with gentiolactone for 24 hours and found no effects on cell viability or growth (Fig. 2B). Gentiolactone significantly suppressed the expression of TNF-α (*Tnf*) and iNOS (*Nos2*), and Cox-2 (*Ptgs2*) at concentrations of 100 µM, 100 µM and 500 µM, respectively (Fig. 3A-C). Moreover, gentiolactone inhibited the LPS-induced accumulation of TNF-α protein (Fig. 3D). Kwak *et al.* reported that gentianine, an anti-inflammatory compound contained in *G. macrophylla radix*, inhibited an LPS-induced increase in IL-6 and TNF-α in rat sera [8]. We used LC-TOFMS to analyze the root extracts of *G. triflora* for gentianine, but did not detect this compound in the most active purified fraction (data not shown). Thus, the anti-inflammatory effect observed in gentian roots was due to the effect of gentiolactone and not gentianine.

**Figure 4:**
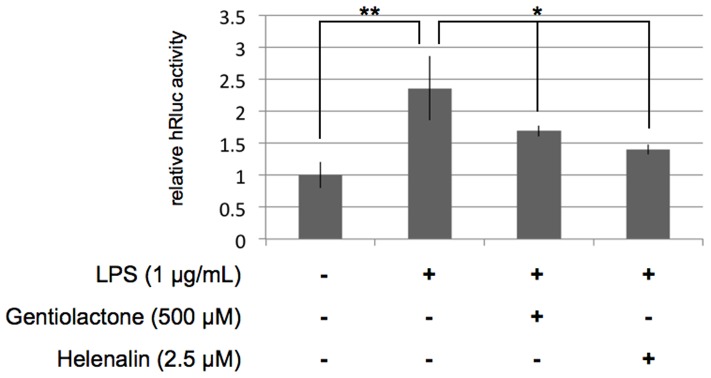
Gentiolactone suppresses transcriptional activity of NF-κB. RAW264.7 cells were transfected with reporter plasmids and cultured with LPS (1 µg/mL) and gentiolactone (500 µM). At 12 hours after LPS stimulation, luciferase activity was measured. Plotted values represent the mean value ± SD from five independent cultures. Helenalin was used as a positive control.

We hypothesized that gentiolactone might interfere with the transcriptional activity of NF-κB. To test this hypothesis, we investigated NF-κB transcriptional activity using the renilla luciferase reporter (hRLuc) gene driven by an NF-κB dependent promoter. We transfected the pNF-κB RE-TK hRluc(F) vector into RAW264.7 cells. At 24 hours after transfection, we added LPS (1 µg/mL) and gentiolactone (500 µM) and measured hRluc activity. The hRluc activity induced by LPS was significantly decreased in the presence of gentiolactone (Fig. 4).

**Figure 5:**
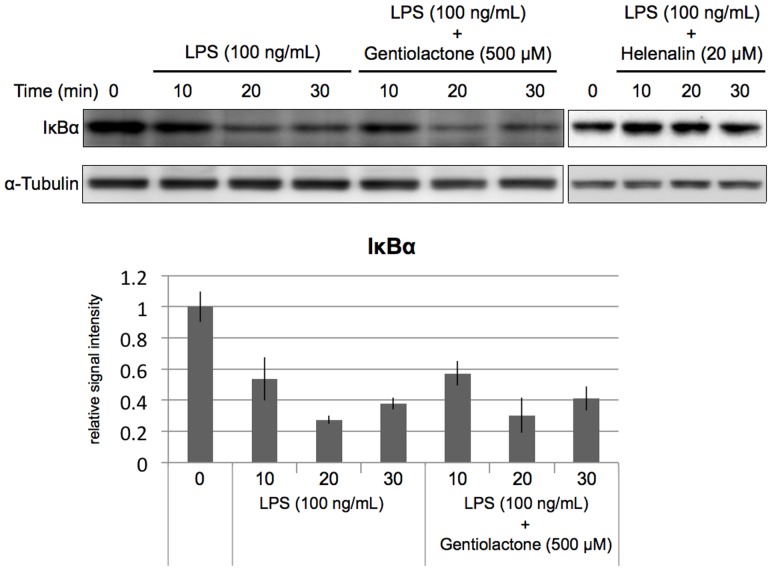
Gentiolactone does not inhibit IκB degradation induced by LPS stimulation. RAW264.7 cells were cultured with LPS (100 ng/mL) and gentiolactone (500 µM) for the indicated periods. Cell extracts from each group were examined by immunoblotting analysis using anti-IκBα antibody. Plotted values represent the mean value ± SD from four independent cultures. Helenalin was used as a positive control.

**Figure 6:**
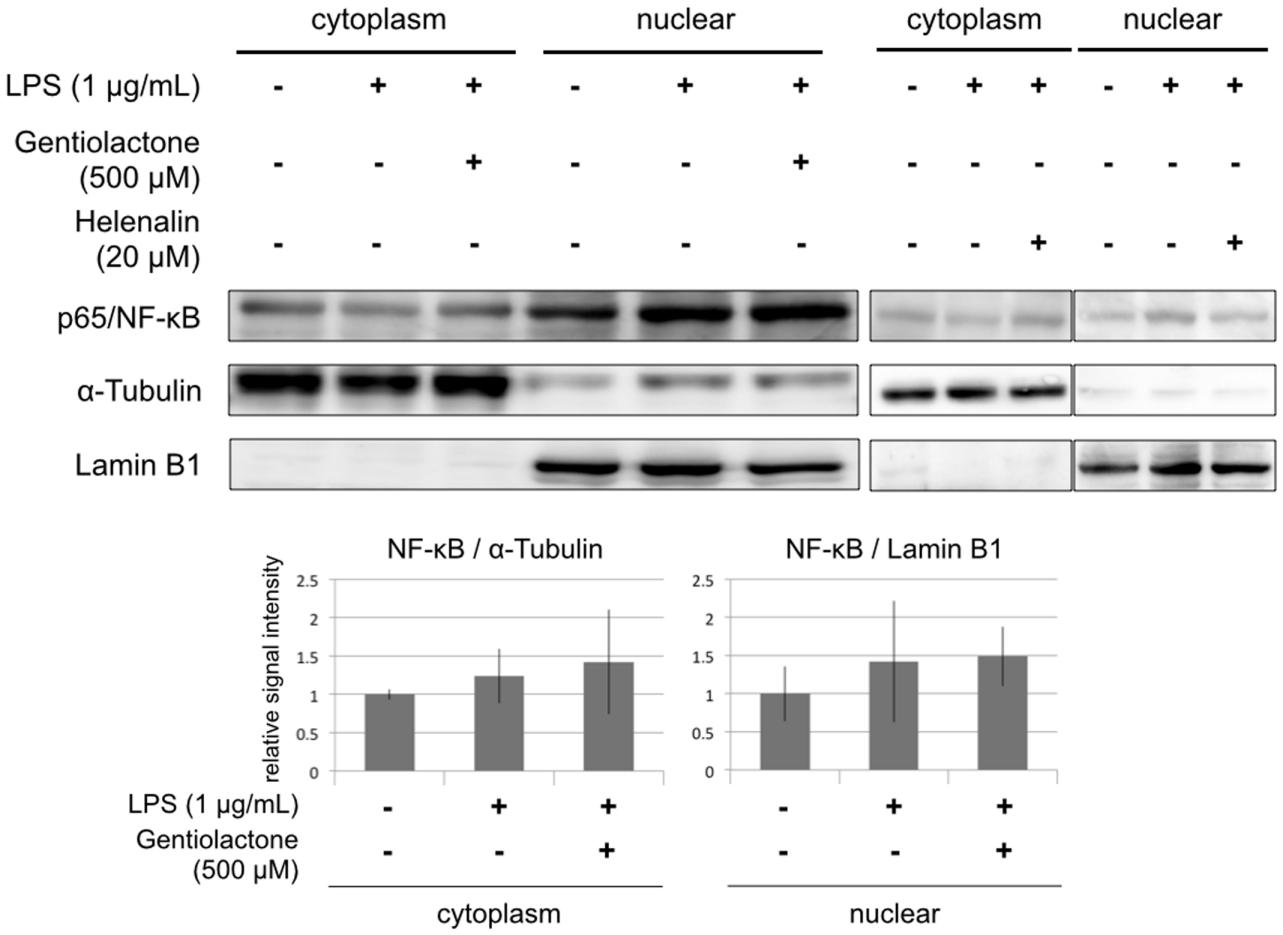
Gentiolactone does not inhibit nuclear transport of NF-κB. RAW264.7 cells were cultured for 1 hour with LPS (1 µg/mL) and gentiolactone (500 µM). The cytoplasmic or nuclear extracts were examined by immunoblotting analysis using anti-p65/NF-κB, anti-α-Tubulin or anti-Lamin B1 antibodies. Plotted values represent the mean value ± SD from four independent cultures. Helenalin was used as a positive control.

**Figure 7:**
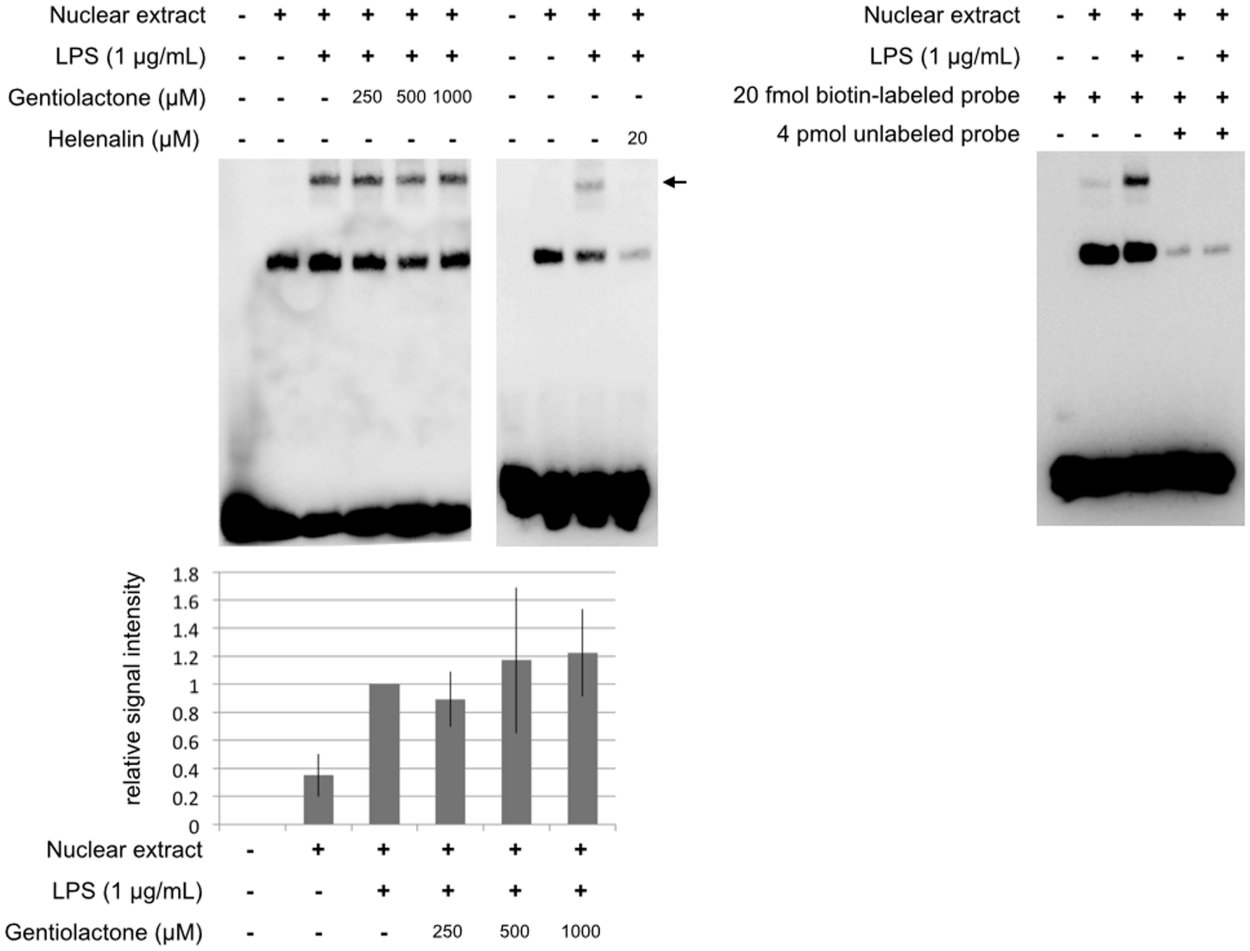
Gentiolactone does not inhibit DNA binding of NF-κB. RAW264.7 cells were cultured for 1 hour with LPS (1 µg/mL) and gentiolactone (250, 500, 1000 µM). Nuclear proteins were extracted and incubated with biotinylated oligonucleotides containing a binding site for NF-κB. Resultant complexes were analyzed by EMSA. The bands indicated by the arrow were quantified. Plotted values represent the mean value ± SD from three independent cultures. Helenalin was used as a positive control.

We examined the effects of gentiolactone on the NF-κB signaling pathway activated by LPS stimulation. LPS stimulation leads to the degradation of the IκB proteins that regulat**e** nuclear NF-κB. The p65:p50 dimer is a member of the NF-κB family and is considered to be part of the canonical pathway. This classic NF-κB dimer is activated by IκB degradation and then translocates to the nucleus and binds to DNA. Gentiolactone did not inhibit IκBα protein degradation (Fig. 5), nuclear translocation of p65/NF-κB protein (Fig. 6), or binding of NF-κB protein to DNA (Fig. 7).

## Discussion

Gentian roots and rhizomes have traditionally been used in Europe and western Asia as herbal medicines because of their anti-inflammatory effects. Most studies of these properties have therefore been performed using European and Chinese gentian roots [7],[19]. In this study, we demonstrated for first time that roots of the Japanese gentian, *G. triflora* (which was first isolated from *G. purpurea* in 1978; [17]), also have anti-inflammatory activity. We identified gentiolactone as the major active compound contained in the root extracts of *G. triflora*. Wang *et al.* (2013) revealed that the inhibitory activity of gentiolactone on nitric oxide production was weaker than gentiopicroside [20]; to our knowledge, this is the only report on the bioactivity of gentiolactone. Here, we showed that gentiolactone prevents accumulation of LPS-induced inflammatory cytokines, TNF-α, and pro-inflammatory enzymes iNOS and Cox-2. Moreover, gentiolactone inhibited the transcriptional activity of NF-κB. Therefore, inhibition of TNF-α, iNOS and Cox-2 mRNA expression and suppression of NF-κB promoter activity by gentiolactone is one of the mechanisms for the anti-inflammatory properties of gentian roots.

The structure of gentiolactone includes a secoiridoid carbon skeleton. Iridoids are naturally occurring substances and are found in many medicinal herbs. They exist mostly as glycosides. Iridoid glycosides seem to be inactive but activated by conversion into hydrolyzed-iridoids by enzymatic hydrolysis of the glycosidic bond. H-aucubin, H-catalpol, H-loganin and H-geniposide have inhibitory effect on LPS-induced TNF-α production, but H-swertiamarin and H-gentiopicroside do not [21]. Previous reports showed that aucubin and geniposide suppress TNF-α production via inhibition of IκB-α degradation [22],[23]. However, gentiolactone suppressed TNF-α production without inhibition of IκB-α degradation or NF-κB nuclear import, suggesting that gentiolactone inhibited TNF-α production in a completely different manner from aucubin and geniposide. As another way of inhibiting TNF-α production, several studies have shown that sesquiterpene, helenaline and parthenolide lactones interact directly with NF-κB to inhibit DNA binding to NF-κB [24]. Anti-inflammatory activity of sesquiterpene lactones are mediated chemically by α,β-unsaturated carbonyl structures, such as α-methylene-γ-lactone or an α,β-unsubstituted cyclopentenone [25]. Helenaline and parthenolide lactones also inhibit IκB-α protein degradation [24]. Sesquiterpene lactones potently inhibit NF-κB, but have toxicity because of their inhibitory effect on DNA and protein synthesis as well as hexokinase activity [26]. In the present study, helenalin was observed to have a cytotoxic effect in RAW264.7 cells: in cultures exposed to 20 µM helenalin for 24 hours, >99.5% of the cells died (Fig. 2B). By contrast, gentiolactone did not have a cytotoxic effect even at concentrations as high as 500 µM. Gentiolactone showed a weaker ability to inhibit LPS-induced TNF-α production than helenalin, but it could suppress TNF-α, iNOS and Cox-2 expression without any cytotoxic effects. Additionally, gentiolactone inhibited NF-κB transcriptional activity but did not inhibit the DNA-binding of NF-κB. These findings indicated that gentiolactone inhibited TNF-α production in a different manner from sesquiterpene lactones. As the inhibitory effect of gentiolactone on TNF-α expression was only evident at 100 µM (21.2 µg/ml), the use of the intact compound as an anti-inflammatory drug might be difficult. However, by determining the active center of gentiolactone, it should be feasible to design derivatives with usable anti-inflammatory activities.

Chronic inflammation is a hallmark of rheumatoid arthritis and atherosclerosis [27],[28]. As NF-κB activates expression of pro-inflammatory cytokines and pro-inflammatory enzymes, so inhibitors of NF-κB are lead compounds for the development of anti-inflammatory drugs. The most common mechanism for NF-κB inhibition is through prevention of IκB-α degradation [29]. However, gentiolactone appeared to inhibit TNF-α expression in a different manner. Identification of the molecular target of gentiolactone and characterization of the mechanism for blocking NF-κB promoter activity will provide new insights for designing novel NF-κB inhibitors.

## Material and Methods

### Plant materials

The roots and rhizomes of *G. triflora* cv. ‘September Blue’ were collected from the field of Iwate Biotechnology Research Center, Iwate, Japan, in June 2012, and were authenticated by Dr. Hideyuki Takahashi, Iwate Biotechnology Research Center. A voucher specimen (GR-Spb-120615) was deposited in the Iwate Biotechnology Research Center.

### Extraction and isolation

The roots were harvested and dried naturally at room temperature. A 10 g sample of dried gentian roots was powdered and then extracted with MeOH (100 ml) under reflux for four hours. The 2.3 g of MeOH extract was subjected to a preparative high performance liquid chromatography (HPLC) system (PLC761; GL Science Inc., Tokyo, Japan). Gentiolactone (12.1 mg) was separated on an InertSustain ODS-3 column (20.0 mm dia. ×250 mm; GL Science Inc.) with gradient elution of water and MeOH (10 to 40% of MeOH in 30 min, 40% of MeOH for 5 min) at a flow rate of 15 mL/min and detection at 240 nm.

### Identification of gentiolactone

Identification of the active constituents was performed by mass spectroscopy (Jeol JMS-700, Jeol Ltd., Tokyo, Japan), nuclear magnetic resonance spectroscopy (NMR) (Jeol EX-400, JeolL Ltd.), UV (Shimazu UV mini 1240, Shimazu Co., Kyoto, Japan) and optical rotation (Jasco DIP1000, Nihon Bunko Co., Tokyo, Japan). All physico-chemical properties, such as fragmentation, molecular formulas, chemical shifts for carbon, UV spectrum and optical rotation ([α]_D_^22^ = -1.4° (*c* = 0.15, MeOH)), were identical with reported data for (±)-gentiolactone (purity >95% by HPLC, Fig. S1) [17].

### Reagents

Swertiamarin (purity >98%) and gentiopicroside (purity >98%) were purchased from Wako Pure Chemical Industries (Osaka, Japan). Helenalin (purity >97%) was purchased from Santa Cruz Biotechnology (Santa Cruz, CA, USA). Helenalin is a sesquiterpene lactone that has been shown to inhibit NF-κB. Because of this known property, we selected Helenalin as a positive control in the present investigation [18]. Lipopolysaccharides (LPS) from *Escherichia coli* O127 and anti-α-tubulin monoclonal antibody were purchased from Sigma-Aldrich (St. Louis, MO, USA). Anti-IκB-α, anti-NF-κB p65 antibodies were purchased from Santa Cruz Biotechnology.

### Cell line and culture

RAW264.7 cells (DS Pharma Biomedical Co., Ltd., Osaka, Japan) were cultured in DMEM containing 10% fetal bovine serum (FBS; Invitrogen, Carlsbad, CA, USA) and Antibiotic Antimycotic Solution (Sigma-Aldrich).

### Liquid chromatography-time of flight mass spectrometry (LC-TOFMS) analysis

Gentiopicroside, swertiamarin or gentiolactone were separated on an InertSustain ODS-3 column (2.0 mm dia. ×150 mm; GL Science Inc.) with gradient elution of 10 mM ammonium acetate (pH 7.8) and MeOH (10 to 40% of MeOH in 30 min) at a flow rate of 0.2 mL/min. Compounds were identified and quantified by LC-TOFMS (Agilent Technologies, Palo Alto, CA, USA) using Agilent Mass Hunter Workstation Software. Quantitative accuracy was verified with known concentrations of reference standard compounds.

### Quantitative real-time PCR

RAW264.7 cells were seeded in 48-well culture plates (1×10^5^ cells/well) and cultured overnight. Then, the cells were cultured in DMEM containing 10% FBS and LPS (100 ng/mL) with gentiopicroside, swertiamarin or gentiolactone for 4 hours. Total RNA was extracted with an RNeasy kit (QIAGEN, Tokyo, Japan) and used to synthesize cDNA with the PrimeScript RT reagent kit (Takara, Shiga, Japan); all kits were used according to the manufacturers' recommendations. Quantitative real-time PCR was performed with the gene specific primers listed in Table S1 and Fast SYBR Green master mix (Applied Biosystems). The qRT-PCR data were normalized against *Actb* (NM_007393).

### ELISA

RAW264.7 cells were seeded in 48-well culture plates (1×10^5^ cells/well) and cultured overnight. The cells were then cultured in DMEM containing 10% FBS and LPS (100 ng/mL) with 500 µM gentiopicroside, swertiamarin or gentiolactone for 8 hours. TNF-α protein levels in the culture media were measured using a Quantikine ELISA mouse TNF-α Immunoassay kit (R&D Systems, Minneapolis, MN).

### Western Blot

To prepare whole-cell lysates, cells were washed twice with ice-cold PBS containing a protease inhibitor cocktail (Roche, Mannheim, Germany), scraped into ice-cold lysis buffer (20 mM Tris-HCl, pH 7.6, 1% NP-40, 150 mM, NaCl, 0.1% sodium deoxycholate, 0.1% SDS and protease inhibitor cocktail). After homogenization, cell debris was removed by centrifugation at 1000×*g* for 10 min. The supernatants were deemed whole lysates. To obtain nuclear and cytoplasmic fractions, we used an NE-PER Nuclear and Cytoplasmic Extraction Kit (Thermo, Rockford, IL, USA). Total protein contents were quantified using Coomassie Plus (Thermo). Proteins in the extracts (50 µg) were separated by 10% SDS–polyacrylamide gel electrophoresis and electrophoretically transferred to Amersham Hybond-P membranes (GE Healthcare, Tokyo, Japan) with NuPAGE transfer buffer (Invitrogen). The membranes were blocked with 2% ELC advance blocking reagent (GE Healthcare) in Tris-buffered saline with 0.1% Tween 20 and incubated with specific primary antibodies; the membranes were then incubated with horseradish peroxidase-conjugated secondary antibodies. Immunoreactive bands were visualized by Lumigen TMA-6 (GE Healthcare) and analyzed using ImageQuant LAS 4000 (GE Healthcare).

### Luciferase reporter assay

We used the pNF-κB RE-TK hRluc(F) vector (Riken BRC, Ibaraki, Japan), which uses the nucleotide sequence of the NF-κB response element found upstream of minimal thymidine kinase (TK) promoter, in the renilla luciferase reporter construct. RAW264.7 cells were seeded in 24-well culture plates (1×10^5^ cells/well) and cultured overnight. The firefly luciferase normalization vector pCMV-Luc and the renilla luciferase reporter construct pNF-κB RE-TK hRluc(F) were co-transfected using Lipofectamine 2000 (Invitrogen) into the RAW264.7 cells, and the transfected cells were cultured for 24 hours. The cells were then cultured in DMEM containing 10% FBS and LPS (1 µg/mL) with or without gentiolactone for 12 hours. The cells were harvested using passive lysis buffer (Promega). Renilla and firefly luciferase activities were determined using a dual luciferase assay system (Promega).

### Electrophoretic mobility shift assay (EMSA)

RAW264.7 cells were seeded in 48-well culture plates (1×10^5^ cells/well) and cultured overnight. Then, the cells were cultured in DMEM containing 10% FBS and LPS (1 µg/mL) with gentiolactone for 1 hour. Nuclear proteins were extracted using an NE-PER Nuclear and Cytoplasmic Extraction Kit (Thermo). A biotin-labeled oligonucleotide (5′ -AGTTGAGGGGACTTTCCCAGGC- 3′) was incubated with 20 µg of nuclear extract at room temperature for 30 min. We used the LightShift Chemiluminescent EMSA Kit (Thermo) and followed the manufacturers' recommendations.

### Statistical analysis

Statistical differences among experimental groups were evaluated by one-way ANOVA and Tukey's post-hoc test. Values are expressed as the mean ± S.D.

## Supporting Information

Figure S1 **Purification of gentiolactone from MeOH extract of** ***G. triflora*** **.** A, The extract (20 mg of the extract in 2 mL 10% MeOH) was separated by InertSustain ODS3 column, and 5 main peaks were found. Peak 1 was collected and identified by LC-TOFMS and NMR as gentiolactone. B, Chromatogram of gentiolactone (60 µg of gentiolactone) purified from *G. triflora*.
(TIFF)

Table S1 **Primers for real-time PCR.**
(DOC)
