# Supplementary figures and images for: Gentiolactone, a Secoiridoid Dilactone from Gentiana triflora, Inhibits TNF-α, iNOS and Cox-2 mRNA Expression and Blocks NF-κB Promoter Activity in Murine Macrophages
Source: PLoS One. 2014 Nov 25;9(11):e113834. doi: 10.1371/journal.pone.0113834 (PMC4244148; doi:10.1371/journal.pone.0113834)

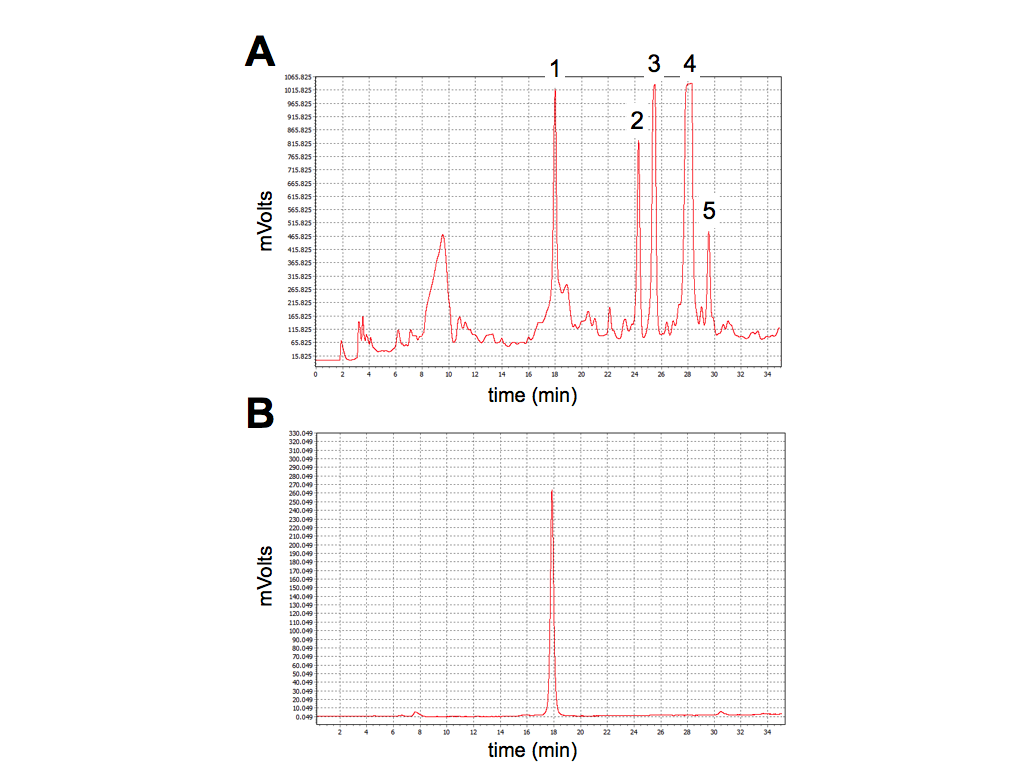

Supplement: Figure S1 — Purification of gentiolactone from MeOH extract of G. triflora . A, The extract (20 mg of the extract in 2 mL 10% MeOH) was separated by InertSustain ODS3 column, and 5 main peaks were found. Peak 1 was collected and identified by LC-TOFMS and NMR as gentiolactone. B, Chromatogram of gentiolactone (60 µg of gentiolactone) purified from G. triflora. (TIFF) [file pone.0113834.s001.tiff]
