# Supplementary material for: Gentiolactone, a Secoiridoid Dilactone from Gentiana triflora, Inhibits TNF-α, iNOS and Cox-2 mRNA Expression and Blocks NF-κB Promoter Activity in Murine Macrophages
Source: PLoS One. 2014 Nov 25;9(11):e113834. doi: 10.1371/journal.pone.0113834 (PMC4244148; doi:10.1371/journal.pone.0113834)
Supplement: Table S1 — Primers for real-time PCR. (DOC) [file pone.0113834.s002.doc]

Table S1 Primers for real-time PCR

| Symbol | Description | Ref seq DNA ID |  |
| --- | --- | --- | --- |
| *Actb* | beta actin | NM_007393 | TGGAATCCTGTGGCATCCATGAAAC |
|  |  |  | TAAAACGCAGCTCAGTAACAGTCCG |
| *Tnf* (TNFα) | tumor necrosis factor | NM_013693 | AGCCCACGTCGTAGCAAACCACCAA |
|  |  |  | ACACCCATTCCCTTCACAGAGCAAT |
| *Nos2* (iNOS) | inducible nitric oxide synthase 2 | NM_010927 | TGGCTCGCTTTGCCACGGAC |
|  |  |  | AAGGCAGCGGGCACATGCAA |
| *Ptgs2* (Cox2) | prostaglandin-endoperoxide synthase 2 | NM_011198 | TGGGTTCACCCGAGGACTG |
|  |  |  | GGGGATACACCTCTCCACCAA |
